# BatchBMD as an Efficient and Accurate Dual-Energy X-ray Absorptiometry Report Generator

**DOI:** 10.3390/diagnostics11122403

**Published:** 2021-12-20

**Authors:** Chun-Hsiang Chan, Wen-Chi Huang, Yi-Chien Lu, Hsing-Fen Hsiao, Wing P. Chan

**Affiliations:** 1 Department of Radiology, Wan Fang Hospital, Taipei Medical University, Taipei 116, Taiwan; d04228002@ntu.edu.tw (C.-H.C.); 106263@w.tmu.edu.tw (Y.-C.L.); 99016@w.tmu.edu.tw (H.-F.H.); 2 Department of Radiology, School of Medicine, College of Medicine, Taipei Medical University, Taipei 110, Taiwan; 3 Department of Computer Science and Information Engineering, National Taiwan University, Taipei 106, Taiwan; chi18000@gmail.com

**Keywords:** dual-energy X-ray absorptiometry, bone mineral density, automated reporting system

## Abstract

Dual-energy X-ray absorptiometry is the gold standard for evaluating Bone Mineral Density (BMD); however, a typical BMD report is generated in a time-inefficient manner and is prone to error. We developed a rule-based automated reporting system, BatchBMD, that accelerates DXA reporting while improving its accuracy over current systems. BatchBMD generates a structured report, customized to the specific clinical purpose. To compare BatchBMD to a Web-based Reporting (WBR) system for efficiency and accuracy, 500 examinations were randomly chosen from those performed at the Taipei Municipal Wanfang Hospital from January to March 2021. The final assessment included all 2326 examinations conducted from September 2020 to March 2021. The average reporting times were 6.7 and 10.8 min for BatchBMD and the WBR system, respectively, while accuracy was 99.4% and 98.2%, respectively. Most of the errors made by BatchBMD were digit errors in the appendicular skeletal muscle index. After correcting this, 100% accuracy across all 2326 examinations was validated. This automated and accurate BMD reporting system significantly reduces report production workload for radiologists and technicians while increasing productivity and quality. Additionally, the portable software, which employs a simple framework, can reduce deployment costs in clinical practice.

## 1. Introduction

Dual-energy X-ray Absorptiometry (DXA) is the gold standard for evaluating Bone Mineral Density (BMD) at the lumbar spine, hip, and forearm, particularly for those with osteoporosis [1], a metabolic disease characterized by increased risks of bone fragility and compression fractures. In clinical practice, DXA is the gold standard for diagnosing osteoporosis, predicting fragility fracture, and monitoring treatment [2,3]. As the population ages, osteoporosis becomes much more prevalent; therefore, a more efficient means of generating BMD reports will be necessary as the demand for DXA examinations increases [4,5,6,7]. DXA report generation is a fully manual process, in that radiologists or technicians must key all the necessary data into the proper fields. The risk level of osteoporosis is then categorized using the DXA images and the *2019 International Society for Clinical Densitometry (ISCD) Official Positions for Adults* [8]. Manual data entry of the BMD data (lumbar spine, hip, and femur) requires a couple of minutes; then the T- and Z-scores are automatically calculated using young adult and age-matched reference populations, respectively [9,10]. The disadvantages of this report-generation process include its time intensiveness, errors in manual entry, misuse of the T- and Z-scores, misuse of the region of interest, dictation errors, and data omissions [11,12,13,14]. These common errors can result in incorrect patient diagnoses and inappropriate pharmacological treatments [15]. Therefore, an automated report-generating system can not only speed the process but improve report accuracy in clinical practice [16].

To solve these challenges, several approaches have been proposed: voice recognition dictation, optical character recognition, and raw data retrieval [9,17,18]. Voice recognition dictation (e.g., Agfa TalkStation) and optical character recognition have replaced the manual key process, reducing typing errors; however, neither is 100% accurate, given the verbal nature of the data, miscategorization, and segmentation [10]. In those studies, testing direct access of the raw data from the DXA database, recognition errors were avoided, but T- and Z-scores, the two crucial indicators necessary for evaluating osteoporosis risk, were not always provided in the raw data [9,18]. Calculating T- and Z-scores without certified reference data does not avoid the original pitfalls—incorrect diagnoses and inappropriate treatments—and the cost of third-party software will increase the overall cost of system deployment. An inexpensive means of automatically generating an efficient and accurate report must be developed.

This study aimed to develop an automated reporting system, BatchBMD, that accelerates report generation while improving accuracy. To accommodate the variety of report formats required in clinical practice, BatchBMD will allow users to customize report templates.

## 2. Materials and Methods

### 2.1. Software Design

In this study, we designed BatchBMD to generate formatted BMD reports using input from a Lunar Prodigy DXA scanner (Lunar Prodigy; GE Healthcare, Madison, WI, USA) and its software, enCORE (version 15, SP1; GE Healthcare, Chicago, IL, USA). enCORE was used to measure BMD at the lumbar spine, femur, and forearm, perform a Lateral Vertebral Assessment (LVA), and determine total body composition. All data and images were processed by enCORE and stored in the Picture Archiving and Communication System (PACS) once the technician reviewed the scan. The data stored in the PACS included regular Digital Imaging and Communications in Medicine (DICOM) reports (image + text) and Structure Reports (SRs; text + XML).

**Figure 1:**
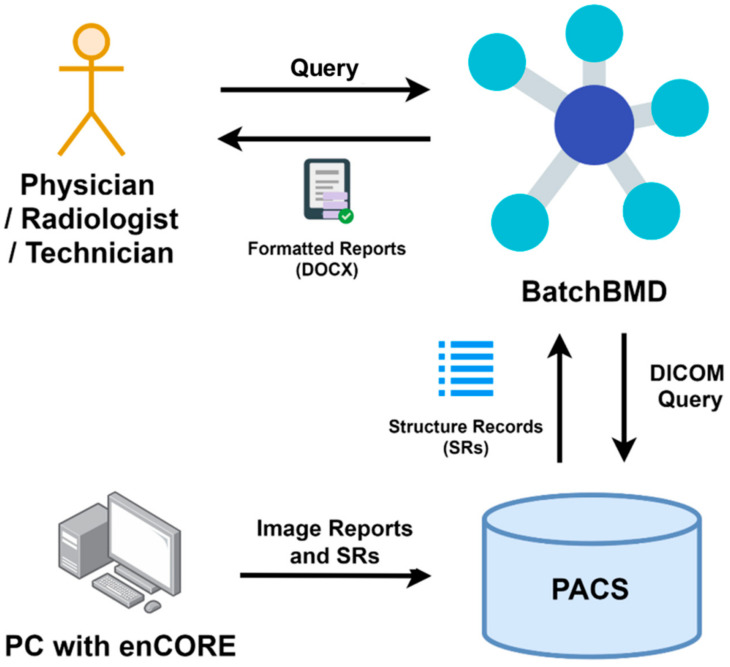
Data flow diagram.

**Figure 2:**
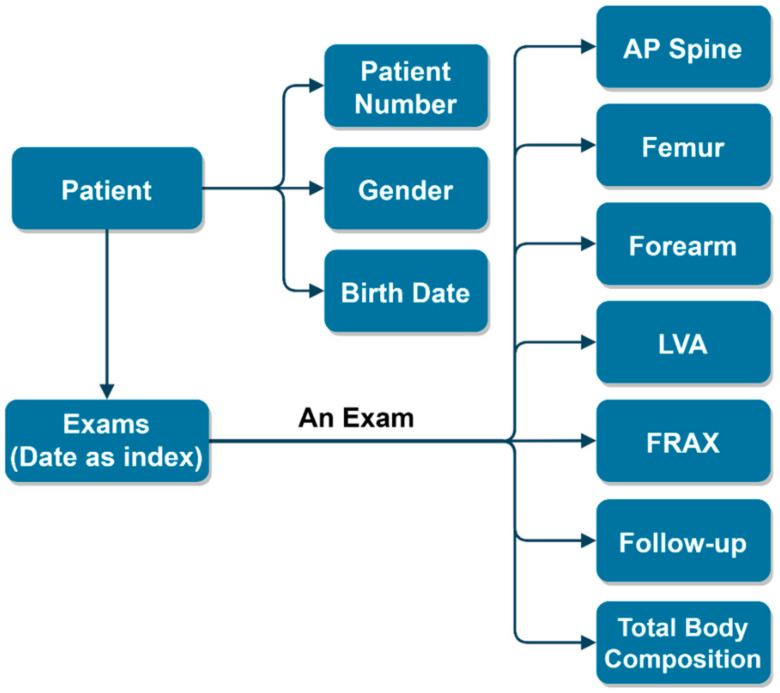
Data structure of the *Patient* object in BatchBMD.

The required data were extracted using the DICOM query/retrieve function to search for and acquire specific SRs. Figure 1 illustrates the data flow and entity relationships. The user can search DXA exams by patient ID, exam date, or both. Once a query is submitted, BatchBMD retrieves the SRs from the PACS, which stores SRs from the DXA software, enCORE. It then generates formatted reports based on ISCD guidelines. Finally, the user can store and print formatted reports for clinical use. Using a single patient examination, extracted SRs from PACS can be separated into: (1) Anteroposterior (AP) spine, both femurs, fracture risk analysis, and baseline information; (2) one or both forearms (forearm data can be combined with the spine and femur data in cases where technician manipulation allows it); (3) LVA (optional); and (4) total body composition (optional). Abstractly, in BatchBMD, each patient temporally owns an object called *Patient* after parsing the SRs, and *Patient* contains all the exams in the PACS for the patient that fulfilled the query. Figure 2 shows the data structure of the *Patient* object. After BatchBMD parses all SRs, each patient temporally owns his/her own *Patient* object, which includes patient number, sex, birth date, and all exams. Each exam is indexed using the exam date, and six types of BMD data, as well as total body composition, baseline information, and 10-year fracture risk evaluation (i.e., the Fracture Risk Assessment Tool (FRAX) score), are included. Finally, BatchBMD extracts the necessary data from each *Patient* object and exports a rule-based formatted report that complies with the 2019 official position of the ISCD [8].

Our institution utilizes five templates for reporting BMD: two for women (postmenopausal/not postmenopausal, coding to T1/Z1), two for men (aged over/under 50 years old, coding to T2/Z2), and one for total body composition. All templates can be user-customized at any time. Conforming with our institution’s software environment, BatchBMD was designed in C# using Visual Studio 2017, and it was executed as a portable Windows application.

### 2.2. Data Acquisition

Five hundred DXA reports from 500 participants scanned from January 2021 to March 2021 were used to develop and evaluate BatchBMD. Another 2326 scans, obtained from September 2020 to March 2021, were enrolled for validation. The enCORE software was used to manipulate the scans, then calculate BMD; T- and Z-scores at the lumbar spine, both femurs (total), and one or both forearms; LVA; total body composition; FRAX score; and baseline information according to DXA report (Figure S1). Of these, the LVA, total body composition, and baseline information were optional. Once all DXA scans were complete, one of three experienced technicians performed the following tasks in order: (1) manually upload the image report and SRs to the PACS, (2) after all scans are complete (once or twice per workday), use BatchBMD to generate formatted BMD and whole body composition reports as Word (.docx) files (Figure S2), (3) review and complete the diagnosis on the formatted reports, and (4) submit reviewed reports to the radiological information system.

### 2.3. Software Evaluation

**Figure 3:**
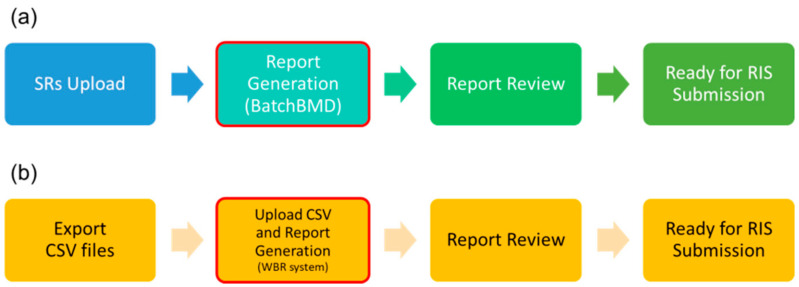
Reporting time computation. (**a**) The report flow for BatchBMD; (**b**) The report flow for a WBR system.

We used the smaller dataset (500 exams) to evaluate whether BatchBMD achieved any clinical improvements and to compare it with a Web-based Reporting (WBR) system. The latter required technicians to manually export several .CSV files from enCORE and upload each to the website; then, the reporting system parsed the .CSV files and exported formatted reports. The uploaded .CSV files contained the raw data, representing the basic information for multiple patients, as well as their BMDs, T- and Z-scores, and selected LVA data (average vertebrae height and anterior, middle, and posterior ratios). Reporting accuracy and average reporting time, with their 95% confidence intervals, were used to evaluate the two systems. Accuracy was calculated using the following formula:

(1) $$Accuracy=\frac{TP}{n}\times 100\%,$$

where *TP* represents the true positive, referring to the number of exported reports without errors by BatchBMD or WBR, and *n* represents the total number of exported reports by BatchBMD or WBR. If accuracy was 100%, it meant that all auto-filled items within exported reports were correct. The reporting time starts at the moment a scan and its SRs are submitted to the PACS for BatchBMD or when .CSV files are exported (WBR system), and it ends at the moment a radiologist finishes reviewing the report, and the report is ready for Radiological Information System (RIS) submission. The processing time of BatchBMD and WBR is only calculated within red-outline blocks (Figure 3). Accuracy significantly differed statistically (paired-sample *t* test, *p* < 0.05) between the two methods. All systematic errors were rectified; then, BatchBMD was validated using the additional set of exams.

## 3. Results

Overall accuracy was better when using BatchBMD. Errors found during evaluation (500 scans) are described in Table 1. The most common error was in the least significant digit of the Appendicular Skeletal Muscle Index (ASMI) in body composition and body composition assessment. On the other hand, the most common error found in the WBR reports was in the LVA, which screens for vertebral compression fractures. The accuracies of BatchBMD and the WBR system were 99.4% and 98.2%, respectively, a statistically significant difference (paired-sample *t* test, *p* < 0.001). After rectifying the least significant digit, the overall accuracy of 2326 examinations was 100%.

**Figure 4:**
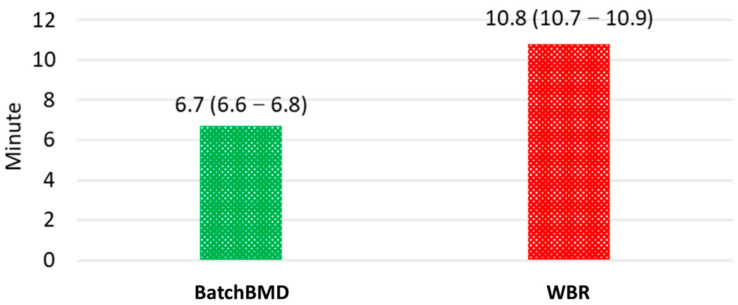
Average (95% confidence interval) reporting time for the two systems.

The efficiency was also better with BatchBMD. The average (95% confidence interval) reporting times for both systems across the 500 scans are shown in Figure 4. Reports using BatchBMD required 6.7 (6.6–6.8) minutes, whereas those using the WBR system required 10.8 (10.7–10.9) minutes. Average software processing times were 1.6 (1.58–1.62) seconds and 0.25 (0.24–0.26) seconds, respectively, a statistically significant difference (paired-sample *t* test, *p* < 0.001). Because BatchBMD needed to obtain the SRs from the PACS, it took a longer software processing time.

Figure S2a shows the BMD reporting template for postmenopausal women, and Figure S2b shows the reporting template for total body composition. Each template shows which data are auto-filled according to Figure S1a–f, and each allows users to copy, paste, or delete items to customize the template.

## 4. Discussion

In this study, we designed an automated DXA reporting system, BatchBMD, to increase the efficiency of data processing while also increasing the accuracy of the data presented in BMD reports. By directly accessing the SRs, the calculated T- and Z-scores and other necessary fields were retrieved for report generation, minimizing the risk of calculation errors from uncertified reference data. This framework could simplify the complexity of DXA data processing. Furthermore, the report template concept used in this system permitted the creation of reports that complied with 2019 ISCD and World Health Organization guidelines [8,19]. BatchBMD is a portable software with minimal deployment costs that provides customizable report templates and thus the flexibility to meet the needs of various applications in clinical practice.

To accelerate and facilitate report generation, others have developed automated reporting systems that can drastically reduce time consumption and error rates [9,10,18,20,21,22]. Tsai, et al. [9] developed an open-source method solely for the Hologic Delphi DXA system, reporting that the average time for processing BMD data was 10.6 s with an accuracy of 98.8%, representing a significant improvement over manual processing (average time, 58.1 s; accuracy, 93.7%). Baker, et al. [21] constructed DXA^2^ to automatically extract data from DXA results, achieving greater efficiency (2.9 s per extraction) and accuracy (100%). DXA^2^ can be deployed with three major DXA systems (GE Healthcare, Hologic, and Norland at Swissray) of several versions; however, it lacks data preprocessing (e.g., data exclusion and data selection) and assessments of osteoporosis risk. Our system combines the advantages of these two methods. BatchBMD directly accesses DXA data and constructs a formal report that includes all the required fields described in the *2019 ISCD Official Positions for Adults* [8] and by Kanis and the WHO Study Group [19]. In all, 41 data fields are specified, and all are included in the PACS and were available to both reporting systems.

The enCORE software calculates T- and Z-scores using internal reference values; however, we found that the SRs it exports contained several data problems: (1) Given any single exam, the least significant digit in patient height or BMI can differ between the SR DICOM tag and the DICOM report; (2) the SR does not contain the ASMI; and (3) when a technician exports an edited report and SR to the PACS, the exam is duplicated, possibly resulting in an incorrect review. The first problem was solved by extracting the raw data for height from the SQL database used by enCORE, producing the value given in the DICOM report. Exam duplication was solved by directing BatchBMD to automatically find the latest value from the SRs, thus generating a report using the edited data. As a result, the accuracy of the modified BatchBMD, used with the larger dataset, was 100% because all the systematic errors were fixed.

The greater efficiency and accuracy of BatchBMD compared to the WBR system was achieved by adopting SRs for retrieving DXA results. Iv et al. [18] reported that using DICOM to query and search the SRs for specific patient data could reduce error rates (e.g., patient mismatches and incorrect values) and shorten time consumption. These improvements were achieved because SRs use XML, a hierarchical and structured format for storing data, and this uniform machine-readable format facilitates exporting data to specific report templates [23]. Others have shown that radiologists and technicians find SRs to be easily readable [24]. Krueger, et al. [25] mentioned that using templates for displaying BMD results could shorten reporting time and enhance report quality. Studies have also shown that radiologists and technicians are more satisfied with SRs because of their completeness [26,27]. However, the hierarchical structure can diminish readability and require more time to generate [28,29]. As a result, Kim, et al. [24] adopted a template-based structure report that enhanced readability and clearly depicted the important BMD results. Given its report production and format flexibility, BatchBMD provided the important and necessary BMD results in a template-based structure, enhancing readability while providing a complete report. Additionally, the report template can be defined by the user to comply with the needs of any clinical purpose or use.

BatchBMD provides five additional benefits to those in clinical practice: BMD trending, a 10-year bone fracture probability, report version management, low deployment cost, and a simple framework. Based on ISCD’s 2019 Official Positions for Adults [8] and Kanis, et al. [30], fracture risk prediction (e.g., the 10-year bone fracture probability) is necessary for providing appropriate therapeutic interventions. Follow-up DXA reports (if any) should also be evaluated in view of a previous or baseline report, noting the statistical significance of any differences between the two as a means of monitoring interventions and evaluating periodic screenings. Good report version management can reduce incorrect diagnoses and inappropriate treatments; therefore, BatchBMD accesses only the latest version of the data to avoid providing an incorrect version. Because BatchBMD was developed as a portable software, its deployment cost is low, and the ability to customize its reports adds flexibility for those in clinical practice. Nevertheless, its simple framework can enhance its applicability and practicability because users can easily modify its code or functions to conform to their current system or version.

This study has three limitations. First, BatchBMD was built for a GE system; therefore, its framework might not apply directly to other DXA systems, depending on their SR formats. Second, using BatchBMD with other software versions could create instabilities due to changes in data formatting, leading to unknown errors; however, because it was designed as a simple framework for connecting DXA results, the PACS, and report generation, it is easy to modify and apply to other systems and versions. Third, this study was conducted at a single institution, which could have affected report-generation efficiency; thus, it should be deployed at other institutions, and its accuracy and efficiency should be tested again.

## 5. Conclusions

BatchBMD is an automated and BMD reporting system that is highly efficient and accurate, significantly reducing the amount of reading and dictating required of radiologists and technicians while increasing productivity in generating BMD reports. Additionally, its template-based structure report enhances readability and reduces misunderstandings while shortening the time needed to reach a diagnosis. Its flexibility allows it to fulfill various clinical purposes. Moreover, the portable and automated nature of this reporting system holds deployment costs to a minimum. Its customizable report templates extend its flexibility to a number of uses in clinical practice. In future, we will deploy BatchBMD at other institutions to prove that its feasibility, accuracy, and practicability are generalizable.

## Figures and Tables

**Table 1:** Accuracy of auto-filled items during evaluation (500 scans) of BatchBMD, compared to a Web-based Reporting system.

Auto-Filled Item Type (Number of Items)	Report Accuracy	
	BatchBMD	WBR
Patient profile (6)	100.0%	100.0%
Lumbar spine BMD info (4)	100.0%	99.8%
Femur BMD info (4)	100.0%	100.0%
Forearm BMD (2)	100.0%	100.0%
LVA, vertebral compression fractures (1)	100.0%	98.2%
Lowest T-score (1)	100.0%	99.8%
Densitometric assessment (1)	100.0%	98.2%
Fracture risk analysis (2)	100.0%	N/A
BMD Follow-up (2)	100.0%	N/A
Body composition (11)	99.4%	100.0%
Body composition assessment (7)	100.0%	N/A

Abbreviations: WBR, Web-based reporting; BMD, bone mineral density; LVA, lateral vertebral assessment; N/A, not applicable.

## Data Availability

Data are not publicly available per the study protocol approved by the Institutional Ethical Review Committee of Taipei Medical University.

## Supplementary Materials

The following are available online at www.mdpi.com/article/10.3390/diagnostics11122403/s1, Figure S1: DXA report example: (a) AP spine BMD; (b) Dual femur BMD; (c) FRAX * 10-year probability of fracture; (d) LVA Morphometry; (e) Left forearm BMD; (f) Body composition; Figure S2: Report Templates. (a) the BMD report template; (b) the whole-body composition report template. Data shown in red are auto-filled by the reporting system.

## Abbreviations

BMD	Bone Mineral Density
WBR	Web-based Reporting
DXA	Dual-energy X-ray Absorptiometry
ISCD	International Society for Clinical Densitometry
LVA	Lateral Vertebral Assessment
PACS	Picture Archiving and Communication System
DICOM	Digital Imaging and Communications in Medicine
SR	Structure Report
AP	Anteroposterior
FRAX	Fracture Risk Assessment Tool
RIS	Radiological Information System
ASMI	Appendicular Skeletal Muscle Index
