# Supplementary material for: BatchBMD as an Efficient and Accurate Dual-Energy X-ray Absorptiometry Report Generator
Source: Diagnostics (Basel). 2021 Dec 20;11(12):2403. doi: 10.3390/diagnostics11122403 (PMC8700356; doi:10.3390/diagnostics11122403)
Supplement: Supplementary file 1 [file diagnostics-11-02403-s001.zip › diagnostics-1463846-supplementary.pdf]

Supplementary Materials

(a)

Taipei Municipal Wan Fang Hospital

No.111, Hsing-Long Road,Sec. 3, Taipei 116, Taiwan, R.O.C.,
(02) 2930-7930 ext:1301

**Patient :**				**Patient ID :**	
**Birth Date :**	73.1 years	Indications :	Menopause (Yes), Secondary Osteoporosis, History of Fracture (Adult)	**Exam ID :**	
**Height / Weight :**	158.3 cm 79.8 kg	Treatment :	No	**Measured :**	2020/12/10 □ □ 11:35:40
**Sex / Ethnic :**	Female Asian	Fracture :	FFx1	**M.P. Age :**	50

AP Spine BMD

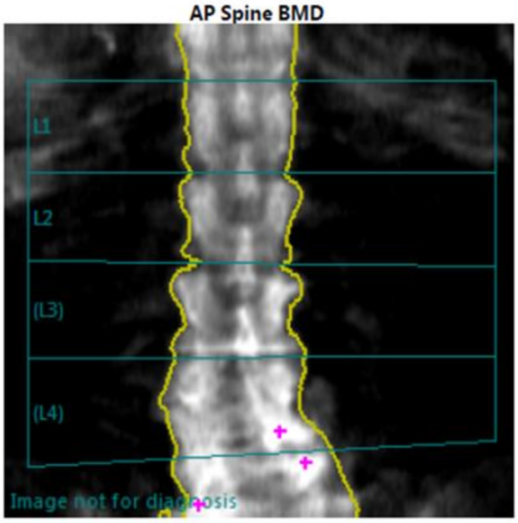

COMMENTS: 51739(J)

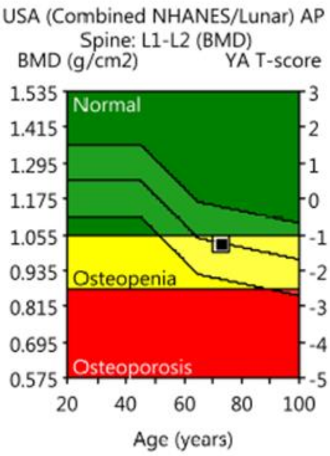

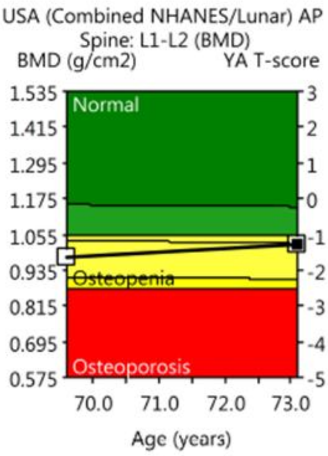

BMD : USA (Combined NHANES/Lunar)

Region	BMD (g/cm2)	Young-Adult T-score
L1	1.017	-1.0
L2	1.024	-1.5
L3	1.175	-0.3
L4	1.428	1.6
L1-L2	1.020	-1.3

BMD Trend : L1-L2

Measured Date	Birth Date □ years□	BMD (g/cm2)	Change vs Baseline (%)	Change vs Baseline (%/yr)
2020/12/10	73.1	1.020	4.1 *	1.1 *
2017/5/24	69.6	0.980	baseline	baseline

(*) Indicates significant change based on 95% confidence interval. (LSC= 0.036 g/cm2 for AP Spine L1-L2); Statistically 68% of repeat scans fall within 1SD (± 0.013 g/cm2 for AP Spine L1-L2); USA (Combined NHANES (ages 20-30) / Lunar (ages 20-40)) AP Spine Reference Population (v113); World Health Organization - Definition of Osteoporosis and Osteopenia for Caucasian Women: Normal = T-score at or above -1.0 SD; Osteopenia = T-score between -1.0 and -2.5 SD; Osteoporosis = T-score at or below -2.5 SD; (WHO definitions only apply when a young healthy Caucasian Women reference database is used to determine T-scores.)
Date created: 2021/10/19 □ □ 03:47:51 15 [SP 1]; Filename: ypt3lq6fp9.dfx; AP Spine; 76:3.00:50.03:12.0 0.00:10.56 0.60x1.05 23.4:%Fat=41.0%; 0.00:0.00 0.00:0.00; Scan Mode: Standard;OneScan; 37.0 µGy

GE Healthcare □ □ 1 □ 1 Lunar Prodigy DF+300285

(b)

Taipei Municipal Wan Fang Hospital

No.111, Hsing-Long Road,Sec. 3, Taipei 116, Taiwan, R.O.C.,
(02) 2930-7930 ext:1301

**Patient :**	[redacted]			**Patient ID :**	[redacted]
**Birth Date :**	[redacted] 73.1 years	Indications :	Menopause (Yes), Secondary Osteoporosis, History of Fracture (Adult)	**Exam ID :**	[redacted]
**Height / Weight :**	158.3 cm 79.8 kg	Treatment :	No	**Measured :**	2020/12/10 □ □ 11:35:40
**Sex / Ethnic :**	Female Asian	Fracture :	FFx1	**M.P. Age :**	50

DualFemur BMD

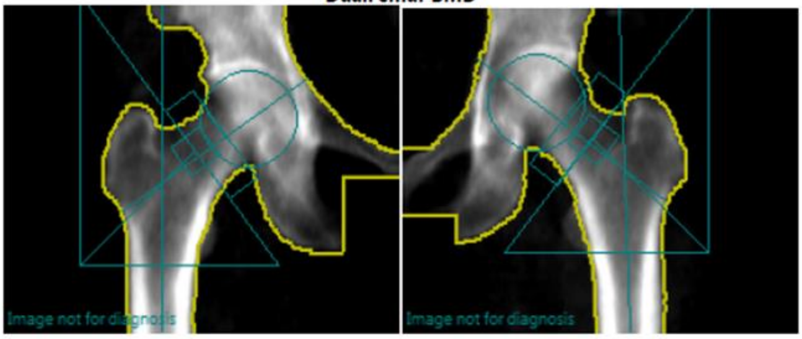

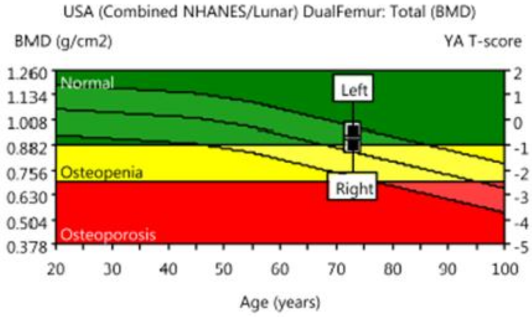

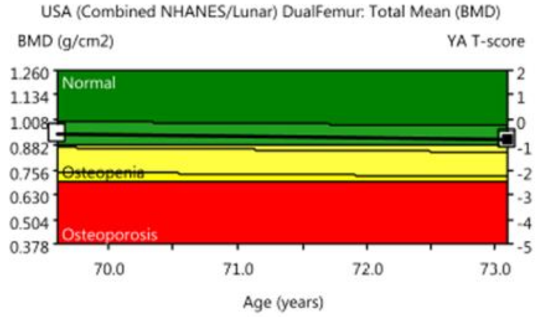

**BMD : USA (Combined NHANES/Lunar)**		
**Region**	**BMD (g/cm2)**	**Young Adult T-score**
Neck Left	0.823	-1.5
Neck Right	0.791	-1.8
Neck Mean	0.807	-1.7
Total Left	0.947	-0.5
Total Right	0.886	-1.0
Total Mean	0.916	-0.7

**Trend : Total Mean**				
**Measured Date**	**Measured Age □ years□**	**BMD (g/cm2)**	**Change vs Baseline (%)**	**Change vs Baseline (%/yr)**
2020/12/10	73.1	0.916	-0.025 *	-2.7 *
2017/5/24	69.6	0.941	baseline	baseline

HAL chart results unavailable

(Right = 100.2 mm) (Left = 102.8 mm)

Right =100.2 mm Avg =N/A mm Left =102.8 mm

COMMENTS: 51739(J)

(*) Indicates significant change based on 95% confidence interval. (LSC= 0.022 g/cm2 for DualFemur Total); Statistically 68% of repeat scans fall within 1SD (± 0.008 g/cm2 for DualFemur Total); USA (Combined NHANES (ages 20-30) / Lunar (ages 20-40)) Femur Reference Population (v113); World Health Organization - Definition of Osteoporosis and Osteopenia for Caucasian Women: Normal = T-score at or above -1.0 SD; Osteopenia = T-score between -1.0 and -2.5 SD; Osteoporosis = T-score at or below -2.5 SD; (WHO definitions only apply when a young healthy Caucasian Women reference database is used to determine T-scores.)

Date created: 2021/10/19 □ □ 03:47:46 15 [SP 1]; Filename: ypt3lq6fp9.dfx; ; Left Femur; 76:3.00:50.03:12.0 0.00:10.20 0.60x1.05 19.6:%Fat=33.6%; 0.00:0.00 0.00:0.00; Neck Angle (deg)= 53; Scan Mode: Standard; 37.0 µGy; Right Femur; 76:3.00:50.03:12.0 0.00:12.06 0.60x1.05 20.1:%Fat=32.4%; 0.00:0.00 0.00:0.00; Neck Angle (deg)= 54; Scan Mode: Standard; 37.0 µGy

(c)

Taipei Municipal Wan Fang Hospital

No.111, Hsing-Long Road,Sec. 3, Taipei 116, Taiwan, R.O.C.,
(02) 2930-7930 ext:1301

**Patient :**				**Patient ID :**	
**Birth Date :**	73.1 years	Indications :	Menopause (Yes), Secondary Osteoporosis, History of Fracture (Adult)	**Exam ID :**	
**Height / Weight :**	158.3 cm 79.8 kg	Treatment :	No	**Measured :**	2020/12/10 □ □ 11:35:40
**Sex / Ethnic :**	Female Asian	Fracture :	FFx1	**M.P. Age :**	50

FRAX* 10-year Probability of Fracture

Based on femoral neck BMD□ DualFemur (Right)

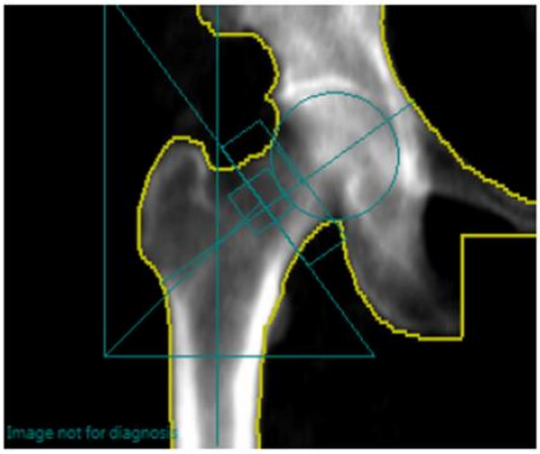

**Major Osteoporotic□**	**15.7%**
**Hip□**	**4.3%**
**Population :**	Taiwan
**Risk Factor :**	Secondary Osteoporosis, History of Fracture (Adult)
**NOF/ISCD Filters:**	None

*FRAX is a trademark of the University of Sheffield Medical School's Centre for Metabolic Bone Disease, a World Health Organization (WHO) Collaborating Centre.

FRAX Version 3.7; The 10-year probability of fracture may be lower than reported if the patient has received treatment.; Major Osteoporotic Fracture: Clinical Spine, Forearm, Hip or Shoulder
Date created: 2021/10/19 □ □ 03:47:47 15 [SP 1]; Filename: ypt3lq6fp9.dfx; Right Femur; 76:3.00:50.03:12.0 0.00:12.06 0.60x1.05 20.1:%Fat=32.4%; 0.00:0.00 0.00:0.00; Neck Angle (deg)= 54; Scan Mode: Standard; 37.0 µGy; Left Femur; 76:3.00:50.03:12.0 0.00:10.20 0.60x1.05 19.6:%Fat=33.6%; 0.00:0.00 0.00:0.00; Neck Angle (deg)= 53; Scan Mode: Standard; 37.0 µGy;

(d)

Taipei Municipal Wan Fang Hospital
No.111, Hsing-Long Road,Sec. 3, Taipei 116, Taiwan, R.O.C.,
(02) 2930-7930 ext:1301

**Patient :**	[redacted]			**Patient ID :**	[redacted]
**Birth Date :**	[redacted] 73.1 years	Indications :	Menopause (Yes)	**Exam ID :**	[redacted]
**Height / Weight :**	158.3 cm 79.8 kg	Treatment :	No	**Measured :**	2020/12/10 □ □ 11:28:52
**Sex / Ethnic :**	Female Asian	Fracture :	FFx1	**M.P. Age :**	50

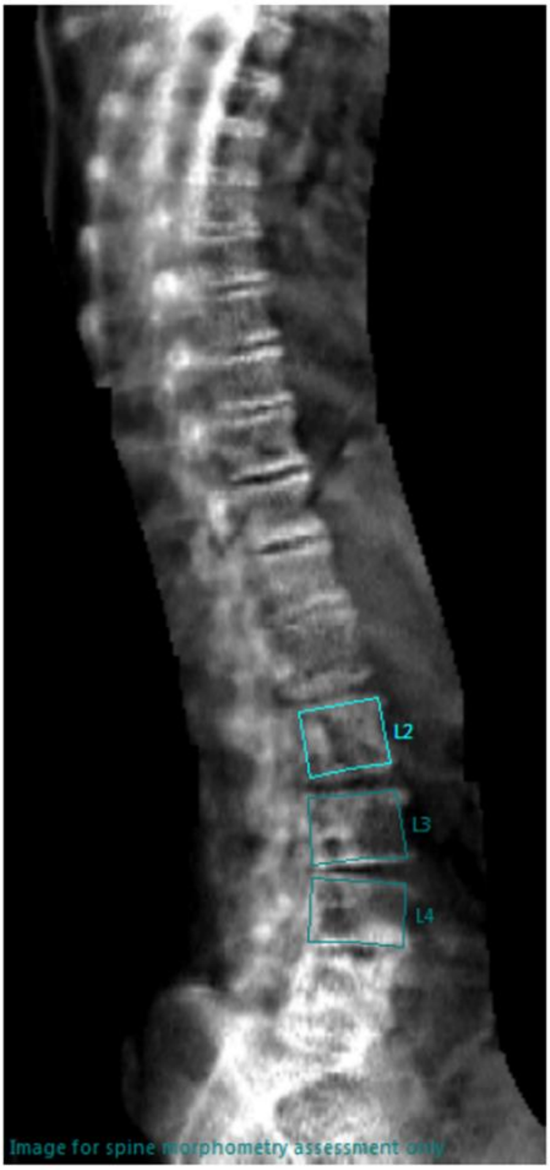

LVA Morphometry

Region	Avg. Ht. □ %□	P/A Ratio □ %□	M/P Ratio □ %□	A/P Ratio □ %□
L2	103	102	95	98
L3	101	99	97	101
L4	96	101	97	99

COMMENTS: 51739(J)

Reference based on L2, L3, and L4; The precision (±1SD) is 1mm for heights and 0.05 for ratios
Date created: 2021/10/19 □ □ 03:45:40 15 [SP 1]; Filename: nit3lq6fp9.dfm; LVA; 76:3.00:22.24:54.0 0.00:17.92 1.20x1.05 26.9:%Fat=24.6%; 0.00:0.00 0.00:0.00; Scan Mode: Standard; 83.0 µGy

(e)

Taipei Municipal Wan Fang Hospital

No.111, Hsing-Long Road,Sec. 3, Taipei 116, Taiwan, R.O.C.,
(02) 2930-7930 ext:1301

**Patient :**				**Patient ID :**	
**Birth Date :**	73.1 years	Indications :	Menopause (Yes), Secondary Osteoporosis	**Exam ID :**	
**Height / Weight :**	158.3 cm 79.8 kg	Treatment :	No	**Measured :**	2020/12/10 □ □ 11:44:06
**Sex / Ethnic :**	Female Asian	Fracture :	FFx1	**M.P. Age :**	50

Left Forearm BMD

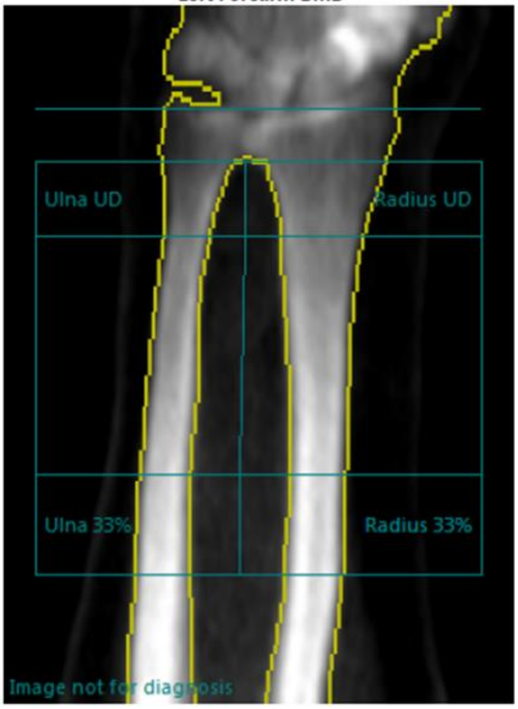

USA (Combined NHANES/Lunar) Left Forearm: Radius 33% (BMD)

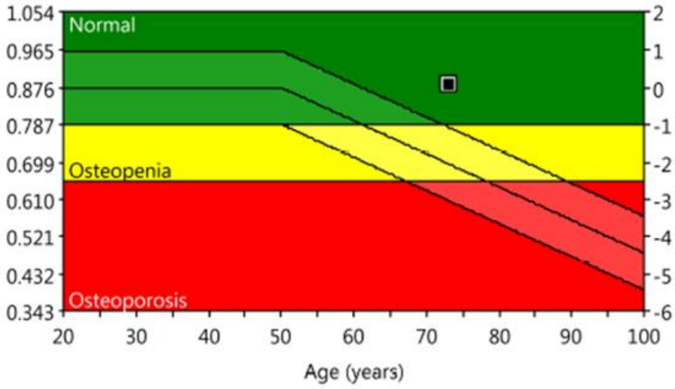

BMD : USA (Combined NHANES/Lunar)

**Region**	**BMD (g/cm2)**	**Young-Adult T-score**
Radius 33%	0.882	0.1

COMMENTS: 51739(J)

Statistically 68% of repeat scans fall within 1SD (± 0.020 g/cm2 for Left Forearm Radius 33%); USA (Combined NHANES (ages 20-30) / Lunar (ages 20-40)) Forearm Reference Population (v113); Lunar calibration in use.; World Health Organization - Definition of Osteoporosis and Osteopenia for Caucasian Women: Normal = T-score at or above -1.0 SD; Osteopenia = T-score between -1.0 and -2.5 SD; Osteoporosis = T-score at or below -2.5 SD; (WHO definitions only apply when a young healthy Caucasian Women reference database is used to determine T-scores.)

Date created: 2021/10/19 □ □ 03:49:03 15 [SP 1]; Filename: wau3lq6fp9.dfa; Left Forearm; 76:0.15:50.03:12.0 0.00:6.20 0.60x1.05 5.0:%Fat=42.3%; 0.00:0.00 0.00:0.00; Forearm Length: 25.1 cm; Scan Mode: Standard;Not seated; 2.0 µGy

(f)

Taipei Municipal Wan Fang Hospital

No.111, Hsing-Long Road,Sec. 3, Taipei 116, Taiwan, R.O.C.,
(02) 2930-7930 ext:1301

**Patient :**				**Patient ID :**	
**Birth Date :**	73.1 years	Indications :	Menopause (Yes), Secondary Osteoporosis	**Exam ID :**	
**Height / Weight :**	158.3 cm 79.8 kg	Treatment :	No	**Measured :**	2020/12/10 □ □ 11:52:17
**Sex / Ethnic :**	Female Asian	Fracture :	FFx1	**M.P. Age :**	50

Total Body Tissue Quantitation

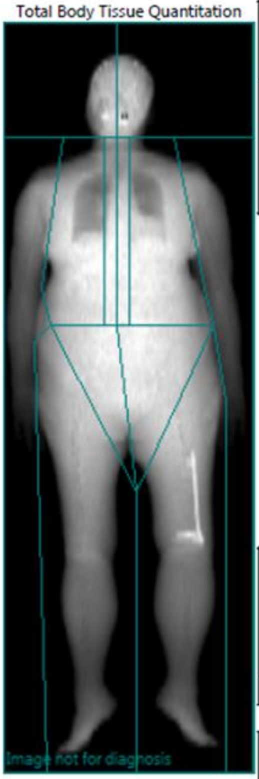

(Enhanced Analysis)

Region	Tissue (%Fat)	Centile	Total Mass (kg)	Fat (g)	Lean (g)	BMC (g)
Arms	42.1	-	7.4	3,030	4,175	216
Legs	37.2	-	26.0	9,380	15,815	766
Trunk	50.4	-	41.7	20,656	20,362	726
Android	55.0	-	6.9	3,747	3,065	53
Gynoid	41.6	-	13.1	5,345	7,511	229
Total	43.9	89	79.7	33,986	43,438	2,279

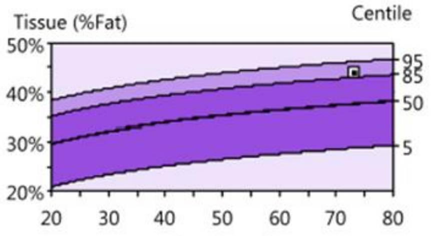

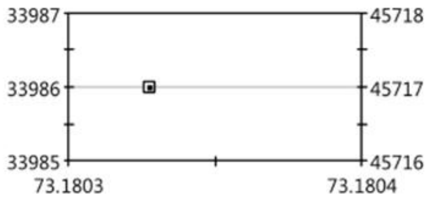

Asia Trend : Total (Enhanced Analysis)

Measured Date	Measured Age □ years □	Tissue (%Fat)	Centile	Total Mass (kg)	Tissue (g)	Fat (g)	Lean (g)	BMC (g)	Fat Free (g)
2020/12/10	73.1	43.9	89	79.7	77,425	33,986	43,438	2,279	45,717

Asia Trend : Distribution (Enhanced Analysis)

Measured Date	Measured Age □ years□	Android (%Fat)	Gynoid (%Fat)	A/G Ratio	Total (%Fat)
2020/12/10	73.1	55.0	41.6	1.32	43.9

Appendicular Skeletal Muscle Index(ASMI): 7.98 kg/m2

Taiwan Reference BMI Classification
Corrected BMI = 31.8 (kg/m2)

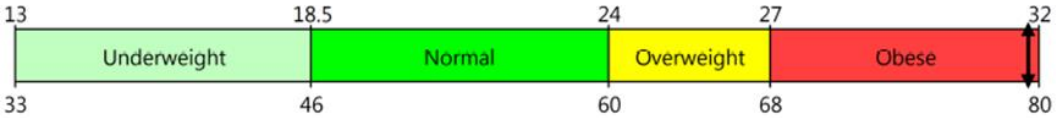

Weight (kg) for height = 158.3 cm

Statistically 68% of repeat scans fall within 1SD (± 0.8 % Fat, ±210 g Tissue Mass, ±520 g Fat Mass, ±610 g Lean Mass for Total Body Total); Asia Total Body Composition Reference Population (v111); Composition Matched for Age
Date created: 2021/10/19 □ □ 03:49:30 15 [SP 1]; Filename: mgu3lq6fp9.dfb; Total Body; 76:0.15:153.04:31.4 0.00:-1.00 4.81x13.01 13.1:%Fat=43.9%; 0.00:0.00 0.00:0.00; Scan Mode: Standard; 0.4 µGy

GE Healthcare □ □ 1 □ 1 Lunar Prodigy DF+300285

Figure S1. DXA report example: (**a**) AP spine BMD; (**b**) Dual femur BMD; (**c**) FRAX* 10-year probability of fracture; (**d**) LVA Morphometry; (**e**) Left forearm BMD; (**f**) Body composition.

(a)

Patient Name: Patient ID: Birth Date: Measured: 2020/12/10

Baseline bone mineral density (BMD) quantification of a 73.1-year-old postmenopausal woman (postmenopausal at 50 years old) at multiple skeletal sites was performed using a dual-energy X-ray absorptiometry scanner (GE-Lunar Prodigy) on 2020/12/10.

Results:

The T-score is calculated using a USA combined (NHANES, ages 20-30/Lunar, ages 20-40) AP spine and femur reference population.

> BMD at the lumbar spine: average BMD at L1-L2 is 1.020 g/cm^2.

(L3 and L4 not quantified due to artifacts / osteophytes / sclerosis / adjacent score > 1) *(not an automatic selection; this must selected by a reporting doctor).*

>BMD at the left proximal femur: femoral neck is 0.823 g/cm^2 and total femur is 0.947 g/cm^2.

>BMD at the right proximal femur: femoral neck is 0.791 g/cm^2 and total femur is 0.886 g/cm^2.

>BMD at the left forearm: radius 33% is 0.882 g/cm^2.

>BMD at the right forearm: radius 33% is N/A g/cm^2.

>VFA: No vertebral compression fractures are noted.

>VFA: Compression fracture at N/A is noted.

>Lowest T-score is -1.8.

Baseline densitometric assessment:

>Densitometric assessment:

-2.5 < T-score < -1.0

>The BMD fulfills the WHO densitometric classification for:

*Low Bone Mass. *(not an automatic diagnosis; this must be filled out by a reporting doctor).*

> Intergrated assessment:

***Osteoporosis due to left femur fracture.** (not an automatic diagnosis; this must be filled out by a reporting doctor).

>Because the T-score is between -1.0 and -2.5, the FRAX™ 10-year probability calculation shows that:

*The risk for any major osteoporosis-related fracture is 15.7%

*The risk for hip fracture is 4.3%

Follow up densitometric assessment:

>The interval significant change of total hip BMD is -2.7%/yr as compared to BMD measured on 2017/5/24.

Recommendations:

This is a baseline study. Follow-up studies are recommended as follows:

-Normal BMD: Consider monitoring (at postmenopause) in 1 year.

-Low bone mass: Consider monitoring in 1-2 years to assess the patient's response to therapy.

-Osteoporosis: Consider monitoring in 1-2 years to assess the patient's response to therapy.

Notes:

-T-score (mean value for young healthy individuals) is applied only to postmenopausal women or men at least 50 years old.

-Z-score (mean value for those of the same age) is applied only to premenopausal women, children, or men younger than 50 years.

-T-score Normative Database: A uniform Caucasian non-race-adjusted female normative database for both women and men of all ethnic groups, based on the 2019 ISCD Official Position.

-WHO definitions apply only when a reference database of young healthy Caucasian women is used to determine T-scores.

(b)

Quantification of total body tissue and whole body composition analysis were performed using a dual-energy X-ray absorptiometry scanner (DXA; Prodigy, GE-Lunar, USA).

Findings:

1. Percentage of fat tissue across the entire body is 43.9%.
2. Total body mass is 79.7 kg which is composed of 33.986 kg fat, 43.438 kg lean mass, and 2.279 kg BMC (bone mineral content).
3. Fat distribution is predominantly Android (55.0%) and Gynoid (41.6 %). The A/G ratio is 1.32.
4. Body mass index (BMI) is 31.8.
5. Handgrip strength is ____ kg. *(this must be filled out by a reporting doctor)*
6. Appendicular skeletal muscle index (ASMI) is 7.98 kg/m^2.

Assessment:

1. The body status as indicated by BMI is Moderate obesity.

Remarks:

BMI indicates body status as follows: <18.5 is underweight, 18.5-<24 is normal, 24-<27 is overweight, 27-<30 is mild obesity, 30-<35 is moderate obesity, and ≧35 is marked obesity (Criteria from the Ministry of Health, Taiwan).

2. The percentage of fat tissue in the entire body is within the 89th percentile using an Asian reference population.

3. Handgrip strength is normal. *(this must be selected by a reporting doctor)*
   Handgrip strength is low.

Remarks: ASIAN WORKING GROUP OF SARCOPENIA(AWGS)

Low handgrip strength: <22.4 kg for men, <14.3 kg for women.

4. Normal muscle mass. *(this must be selected by a reporting doctor)*

Remarks: ASIAN WORKING GROUP OF SARCOPENIA(AWGS)

Low muscle mass: ASMI (RSMI or ASM/ht^2) <7.0 kg/m^2 for men, <5.4 kg/m^2 for women.

Integrated assessment: *(this must be selected by a reporting doctor)*

No sarcopenia.

Sarcopenia.

Sarcopenia probable.

Recommendations:

1. This is a baseline study, and a follow-up study is recommended when body weight changes 5% to 10%.
2. In clinical practice, a patient with low handgrip strength and normal muscle mass should be assessed for causes, and intervention should be initiated.

Figure S2. Report Templates. (**a**) the BMD report template; (**b**) the whole body composition report template. Data shown in red are auto-filled by the reporting system.
